# Gassericins from *Lactobacillus Paragasseri* K7: Preparative Isolation and Discovery of Dose-Dependent Anti-Inflammatory Effects

**DOI:** 10.1007/s12602-025-10910-x

**Published:** 2026-02-07

**Authors:** Humna Liaqat, Diana Paveljšek, Jernej Oberčkal, Gabriela Ambrožová, Kristýna Turková, Lukáš Kubala, Adrijana Leonardi, Igor Križaj, Bojana Bogovič Matijašić

**Affiliations:** 1 Department of Animal Science, Biotechnical Faculty, University of Ljubljana, Domžale, 1230 Slovenia; 2 Department of Biophysics of Immune System, Institute of Biophysics of the Czech Academy of Sciences, Brno, Czech Republic; 3 International Clinical Research Center, St. Anne’s University Hospital Brno, Brno, Czech Republic; 4 Faculty of Science, Institute of Experimental Biology, Masaryk University, Brno, Czech Republic; 5 Department of Molecular and Biomedical Sciences, Jožef Stefan Institute, Ljubljana, 1000 Slovenia

**Keywords:** Gassericins K7A, Gassericins K7B, *Lactobacillus paragasseri* K7, Bacteriocins, Anti-inflammatory activity, Purification, Immunomodulation

## Abstract

Gassericins K7 are heterodimeric bacteriocins produced by *Lactobacillus paragasseri* K7. Their biological characterisation has been limited by challenges in obtaining these compounds in sufficient purity and quantity. In this study, we used a preparative isolation and purification workflow for gassericins K7, combining ammonium sulphate precipitation, Amberlite XAD-16 extraction, and reversed-phase chromatography. Mass spectrometry confirmed the presence of all four peptide components GasK7A α, GasK7A β, GasK7B α, and GasK7B β in the pooled preparation, including GasK7A β, identified here for the first time in natural isolates of this strain. The purified bacteriocin preparation exhibited strong antibacterial activity against indicator strain *Latilactobacillus sakei* NCDO 2714. Cytotoxicity testing in RAW 264.7 macrophages revealed minimal effects at concentrations up to 50 µg/ml, with only a modest reduction in cell viability observed at 100 µg/ml. Subsequent evaluation of immunomodulatory properties demonstrated a clear dose-dependent anti-inflammatory effect in LPS-stimulated macrophages. Treatment with gassericins K7 reduced nitric oxide (NO) production, downregulated inducible nitric oxide synthase (iNOS) expression, and suppressed secretion of the pro-inflammatory cytokines TNF-α and IL-6. Importantly, exposure of macrophages to gassericins alone did not elicit any inflammatory response. These findings provide the first evidence of anti-inflammatory activity of purified gassericins K7 in eukaryotic immune cells and highlight their potential as postbiotic components or biotherapeutic candidates for inflammatory disorders. The preparative method used in this study enables the production of gassericins K7 in quantities sufficient for advanced in vitro and in vivo investigations, facilitating future development of bacteriocin-based therapeutics.

**Supplementary Information:**

The online version contains supplementary material available at 10.1007/s12602-025-10910-x.

## Introduction

The intestinal tract is a dynamic and complex ecosystem in which the host’s tissues coexist with a dense and diverse community of microorganisms, collectively referred to as the gut microbiota. This microbial community plays a fundamental role in maintaining intestinal homeostasis, supporting immune system development, facilitating nutrient metabolism, and preserving epithelial barrier integrity. Disruption of this balance — a condition known as dysbiosis — is increasingly recognized as a contributing factor in the pathogenesis of various inflammatory diseases of the gut, including inflammatory bowel disease (IBD), Crohn’s disease, and ulcerative colitis [1–3]. Commensal microbiota-derived metabolites, such as short-chain fatty acids (SCFAs) and tryptophan-derived indole compounds, are important in reducing gut inflammation and restoring balance [1]. The improvement of IBD by lactic acid bacteria, a well-known source of probiotic strains, and their metabolites has been documented in several in vitro and in vivo studies [4–6]. In addition to probiotics and live biotherapeutics, there is increasing interest in postbiotics — preparations containing inanimate microorganisms and/or their components, often in combination with bioactive bacterial metabolites such as SCFAs, exopolysaccharides, vitamins, and bacteriocins [7, 8].

Recent advances in microbiome research have drawn attention to the potential therapeutic role of microbiota-derived molecules, including bacteriocins — ribosomally synthesized anti-microbial peptides produced by various bacterial species [9]. Traditionally studied for their role in microbial competition and food preservation, they are now being recognised as potential modulators of gut microbiota composition and intestinal immunity. By selectively inhibiting pathogenic or pro-inflammatory bacteria, while sparing beneficial commensals, bacteriocins may help restore microbial balance and mitigate inflammation [10–12]. In addition, recent evidence suggests that some bacteriocins interact directly with the host immune response and, beyond their anti-microbial activity, may have anti-inflammatory properties and promote the expression of tight junction proteins (e.g., ZO-1, occludin, claudin-1), increase mucin production, and improve intestinal epithelial barrier integrity, which helps prevent further inflammation and tissue damage [10–14]. Bacteriocins reduce the production of pro-inflammatory cytokines (e.g., TNF-α, IL-6, IL-1β) and inhibit inflammatory signaling pathways (MAPK, NF-κB), leading to reduced inflammatory responses in the gut [10, 11, 13–15]. Immunomodulation by bacteriocins has been demonstrated in in vitro studies with various cell models [16]. The low toxicity and anti-inflammatory properties of bacteriocins, including nisin ZP, pediocin, CBP22, RSQ04, and plantaricin A, with minimal adverse effects in animal models and significant cytokine reduction in various cell-based assays have been demonstrated [17–20].

*Lactobacillus (L.) paragasseri* K7, isolated from infant feces, produces gassericins K7A (GasK7A) and gassericins K7B (GasK7B), both class IIb two-component bacteriocins (GasK7A α, GasK7A β, GasK7B α and GasK7B β). The bacterial strain and its bacteriocins have already been well characterised in our in vitro and in vivo studies [21–27]. Nilsen and colleagues (2020) identified *L. paragasseri* K7 as the most potent out of the strains scanned against *L. iners*, the bacterium associated with vaginal dysbiosis [28]. They cultured *L. paragasseri* K7 and isolated three anti-microbially active peptides, GasK7A α, GasK7B α and GasK7B β, while GasK7A β could not be obtained directly and was instead produced heterologously in *E. coli* [28].

In this study, we sought to improve the isolation of gassericins K7A and K7B, both class IIb two-component bacteriocins produced by *L. paragasseri* K7, by preparative chromatography and to investigate their immunomodulatory properties using RAW 264.7 macrophages.

## Materials and Methods

### Production of Bacteriocins

Gassericins K7A and K7B were produced by *L. paragasseri* K7 (ZIM 105, Slovenian Collection of Industrial Microorganisms, WFCC #810, Ljubljana, Slovenia; CCM 7710, Czech Collection of Microorganisms, Brno, Czech Republic). For inoculum preparation, 2 ml of an overnight MRS (Merck-Millipore, Germany) culture of *L. paragasseri* K7 was transferred into 200 ml of modified MRS broth and incubated for 18 h at 37 °C under anaerobic conditions using Genbox (Biomerieux, France). Modified MRS broth contained a reduced amount of yeast extract (5 g/L; Merck, Germany) and glucose (10 g/L; Roth, Germany) compared to the conventional formulation [29]. Yeast extract and Tween^®^ 80 were purchased from Biolife (Italy), the other ingredients from Merck-Millipore (Germany). Bacteriocins were produced in a KLF 2000 bioreactor (3.7 L; Bioengineering AG, Wald, Switzerland) at a pH of 5.75, 37 °C and an agitation speed of 350 rpm as previously described [27]. Growth was monitored by measuring the optical density at 600 nm (OD_600_) every 6 min using Shiva II software (Bia, Slovenia). The sterilised medium (1800 ml) was inoculated with 200 ml of the 18-h *L. paragasseri* K7 culture, and the vessel was flushed with N_2_ for 15 min. Fermentation broth samples were taken periodically to test the antibacterial activity, and the process was stopped at the early stationary phase. The broth was centrifuged (15,000 × g, 15 min, 4 °C) to remove bacterial cells, microfiltered (0.4 μm), and stored at −20 °C.

### Isolation of Bacteriocins

Purification was performed according to the protocol of Nilsen et al. [28] with modifications. 62 g ammonium sulphate (Merck, Germany) per 100 ml of cell-free supernatant (CFS) was added to obtain a solution with a final concentration of 45% (w/v) (80% saturation), stirred for 1 h and incubated overnight at 4 °C. The suspension was centrifuged (15000 × g, 20 min, 4 °C), and pellets were resuspended in distilled water to 20% of the initial CFS volume. Amberlite XAD-16 resin (Supelco, Germany) (11 g per 100 ml of initial CFS) was prewashed with methanol, rinsed extensively with water, and added to the suspension. After stirring for 1 h, the resin was separated by filtration, washed twice with 40% ethanol (Merck, Germany), and eluted with 95% isopropanol (Sigma-Aldrich, Germany) containing 0.1% trifluoroacetic acid (TFA) (Merck, Germany). We repeated the elution up to 5-times to elute as much of active material as possible.

The eluates were diluted 4–5-times with 0.1% TFA in water to reduce the solvent concentration, filtered (0.2 μm), and applied to a YMC Triart Prep C18-S column (250 × 10 mm, 15 μm) on a Knauer Azura FPLC system (Germany). Elution was performed with a gradient of mobile phase A (20% isopropanol/0.1% TFA (v/v) in water) and mobile phase B (95% isopropanol/0.1% TFA (v/v) in water). Gradient profile: 0–1 min, 0–12% B; 1–41 min, 12–52% B; 41–43 min, 52–100% B; 43–53 min, 100% B; 53–56 min, 100–0% B; 56–66 min, 0% B. The flow rate was 5.5–7 ml/min during loading and 3.5 ml/min during elution. Detection was performed at 200, 214, 226, and 280 nm. The fractions were analysed by SDS-PAGE, analytical RP-HPLC, and mass spectrometry. The anti-microbial activity was assessed using an agar spot test and microdilution assay. Selected active fractions were subjected to re-chromatography using acetonitrile as eluent to improve purity.

### Analytical Reversed-phase High-Performance Liquid Chromatography (RP-HPLC)

Purified samples were diluted with 5% (v/v) acetonitrile (ACN; Merck, Germany) and 0.1% (v/v) TFA in water (mobile phase A) and analysed using a Shimadzu HPLC system (Kyoto, Japan) with a diode array detector and a Phenomenex C8 Widepore Aeris column (3.6 μm, 200 Å, 250 × 4.6 mm; Phenomenex, USA) at 45 °C. The mobile phase B was 95% (v/v) ACN/0.1% (v/v) TFA in water. Gradient: 0–2 min, 0–20% B; 2–32 min, 20–50% B; 32–34 min, 50–100% B; 34–36 min, 100% B; 36–38 min, 100–0% B; 38–48 min, 0% B. Elution was monitored at 214 and 280 nm. The quantification of bacteriocins in the samples (a mixture of gassericins K7) was estimated by comparing the area under the curve of the absorbance peaks measured at 214 nm with those of a known amount of commercially available nisin Z used as a standard (Handary, Belgium).

### Sodium Dodecyl Sulphate-polyacrylamide Gel Electrophoresis (SDS-PAGE)

Fractions containing organic solvents were dried using a MiVac vacuum concentrator (Genevac, UK), resuspended in non-reducing loading buffer, heated to 95 °C for 5 min, and centrifuged (14,000 × g, 2 min). Samples were separated by tricine SDS-PAGE (Bio-Rad, USA) using 4% stacking, 10% spacer, and 15% separating gels [30]. Proteins were stained with 0.25% Coomassie Brilliant Blue R250 (Thermo Scientific, USA) in 30% ethanol/10% acetic acid (Merck. Germany) and destained with the same solution without dye.

### Determination of Antibacterial Activity

Two assays were carried out: agar diffusion assay and microdilution assay with *Latilactobacillus sakei* NCDO 2714. For agar diffusion, lactobacilli from an overnight culture (10⁵–10⁶ CFU/ml) were incorporated into soft MRS agar (0.75% agar-agar) (Merck, Germany) and poured into Petri dishes. 5 µL of the samples were spotted onto the agar surface, allowed to dry, and incubated anaerobically for 16–18 h at 30 °C. The zones of inhibition indicated the activity. For microdilution, the overnight cultures were diluted 1:10,000 in MRS broth (Merck, Germany). The samples (50 µL) were serially diluted twofold in 96-well plates, combined with 150 µL of diluted indicator strain (10⁴ CFU/well), and incubated anaerobically at 30 °C for 16–20 h. Growth was measured at 630 nm (BioTek Cytation 3 plate reader; BioTek, USA). The lowest dilution inhibiting ≥ 50% growth was used to calculate bacteriocin activity units (AU/ml) as previously described [31].

### Mass Spectrometry

Peptide masses were determined using electrospray ionisation quadrupole time-of-flight mass spectrometry (ESI-Q-TOF-MS) on a Compact mass spectrometer (Bruker Daltonics, Germany) equipped with an Apollo II ESI source. Samples were dissolved in 50% (v/v) acetonitrile and 0.1% (v/v) formic acid at 1 pmol/µL and infused directly into the ion source at 0.18 µL/min. External calibration was performed with “ESI Low Concentration TuneMix” (Agilent Technologies, USA) over a range of 300–3000 m/z. The spectra were acquired in positive ion mode using default parameters for intact proteins: capillary voltage 4500 V, dry temperature 180 °C, dry gas 4 L/min, nebuliser pressure 0.4 bar, and in-source CID 80 eV. Data were processed with Bruker qToF Control v6.3.0.5 and analysed in DataAnalysis 6.0 with deconvolution via maximum entropy algorithms.

To identify the primary sequence, trypsin digestion in solution of gassericins was performed, followed by analysis of the tryptic peptides by nano-liquid chromatography coupled with tandem mass spectrometry (nano-LC-ESI-Q-TOF-MS/MS) using the Compact mass spectrometer (Bruker Daltonics, Germany) as previously described [32]. Tandem mass spectra were searched against *L. paragasseri* bacteriocin sequences from UniProt (retrieved 5 Dec 2024) using Mascot v3.0 (Matrix Science, UK). Search parameters: 20 ppm precursor tolerance, 0.6 Da fragment tolerance, two missed cleavages, carbamidomethyl-Cys as fixed, oxidised Met and deamidated Asn/Gln as variable modifications, with an automated Decoy search and 1% target false discovery rate (FDR).

### Cell Culture

RAW 264.7 murine macrophages (ATCC, USA) were cultured in Dulbecco’s Modified Eagle Medium (DMEM; Gibco, USA) supplemented with 10% fetal bovine serum (Thermo Fisher Scientific, USA), L-glutamine, sodium pyruvate, and 100 U/ml penicillin plus 0.1 µg/ml streptomycin (Gibco, USA). Cells were maintained at 37 °C in a humidified 5% CO₂ atmosphere, with media changed every 2–3 days. Subculturing was performed at 80% confluence.

The organic solvents were removed from the pooled FPLC fractions containing gassericins by miVac centrifugal concentration (Genevac, UK). The dried powder was resuspended in 150 mM NaCl buffer and the pH was adjusted to 3 with acetic acid. This stock solution (1.51 mg/ml gassericins K7) was further diluted in DMEM.

LPS from *Escherichia coli* O26:B6 (Merck, Germany) was prepared at 10 µg/ml in 0.1% bovine serum albumin (BSA)/phosphate-buffered saline (PBS). For NO and cytokine assays, cells (10.−17. passage) were seeded at 2.5 × 10⁴ cells/cm² in 24-well plates for 24 h, then treated with 50 ng/ml LPS with or without gassericins K7 (25, 50, or 100 µg/ml). After 24 h, culture media were collected, centrifuged (250 × g, 5 min, 4 °C) to remove cells, and stored at − 20 °C until analyses. The remaining cells on the plates were washed with cold PBS and used for protein expression analysis by Western blotting.

### Cell Viability Assay

Macrophage cell viability was assessed using the thiazolyl blue tetrazolium bromide (MTT) assay (Thermo Fisher Scientific, USA), following a standard and widely used protocol [33, 34]. RAW 264.7 cells were seeded in 24-well plates for 24 h, treated with gassericins K7 (25–100 µg/ml) and/or LPS (50 ng/ml) for an additional 24 h, and incubated with MTT solution (0.25 mg/ml in medium) for 3 h. After removing the supernatant, formazan crystals were solubilised with acidified 10% Triton X-100 in 0.1 M HCl, and absorbance was measured at 570 nm with a 450 nm reference. Viability was expressed relative to untreated controls:

$$\:Viability\:rel.\:to\:control\left(\%\right)=\left(\frac{A_{\mathrm{s}\mathrm{a}\mathrm{m}\mathrm{p}\mathrm{l}\mathrm{e}}}{A_{\mathrm{c}\mathrm{o}\mathrm{n}\mathrm{t}\mathrm{r}\mathrm{o}\mathrm{l}}}\right)\times\:100\;\lbrack33\rbrack$$

### NO Production

NO levels were determined indirectly via nitrite quantification using the Griess assay [33, 34]. Equal volumes of culture supernatant and Griess reagent (Sigma-Aldrich, Germany) were mixed in 96-well plates, incubated for 15 min at room temperature and absorbance measured at 540 nm (SPECTRA Sunrise microplate reader; Tecan, Switzerland). Concentrations were calculated from sodium nitrite standard curves.

### Pro-inflammatory Cytokines

TNF-α and IL-6 levels were quantified in cell culture medium collected after the treatment of RAW 264.7 cells with LPS and gassericins using ELISA kits (Cat. No. 88–7324-88, 88–7064-88, Invitrogen, Thermo Fisher Scientific, USA) according to manufacturer instructions. Results were expressed in pg/ml.

### Western Blotting

Expression of inducible iNOS and arginase-I was evaluated by Western blotting. RAW 264.7 cells were lysed in 1% SDS buffer containing 1% PMSF and protease inhibitor cocktail (PIC; Thermo Fisher Scientific, USA), scraped, sonicated, and heated for 5 min at 100 °C. Protein concentrations were determined with Pierce™ BCA assay (Thermo Fisher Scientific, USA) and adjusted to 1 mg/ml. Samples were mixed with Laemmli buffer, heated for 10 min at 100 °C, and separated on 10% SDS-polyacrylamide gels, running buffer, 70/110 V (Protein Electrophoresis and Blotting Equipment, Bio-Rad, USA). Proteins were transferred to PVDF membranes, transfer type: wet, transfer buffer, 230 mA, 2.5 h (Protein Electrophoresis and Blotting Equipment, Bio-Rad, USA) and probed with primary antibodies against iNOS (Cat. No. 610431, BD Biosciences, USA), arginase-I (Cat. No. 9819, Cell Signaling Technology), and vinculin (loading control; Cat. No. 13901, Cell Signaling Technology, USA). Secondary antibodies were HRP-linked anti-rabbit IgG or anti-mouse IgG (Cat. No. 7074, 7076, Cell Signaling Technology, USA). Bands were visualised with ECL™ detection reagents (Pierce, USA) and quantified by densitometry using ImageJ. Results were expressed in arbitrary units.

### Statistical Analysis

Data are presented as mean ± SD. Statistical analyses for the Griess (NO production) assay and ELISA (IL-6, TNF-α) were performed using one-way ANOVA followed by Dunnett’s multiple comparison test (*p* < 0.05) in GraphPad Prism 6 (GraphPad Software, San Diego, CA, USA). Three independent experiments were conducted. For the MTT assay, cell viability data were analyzed using Kruskal–Wallis test followed by Dunn’s post hoc test in GraphPad Prism 6, comparing each treatment group with the control without LPS. Three independent experiments were performed.

## Results and Discussion

### Production of Gassericins K7 by *L. Paragaseri* K7 and their Purification

**Fig. 1:**
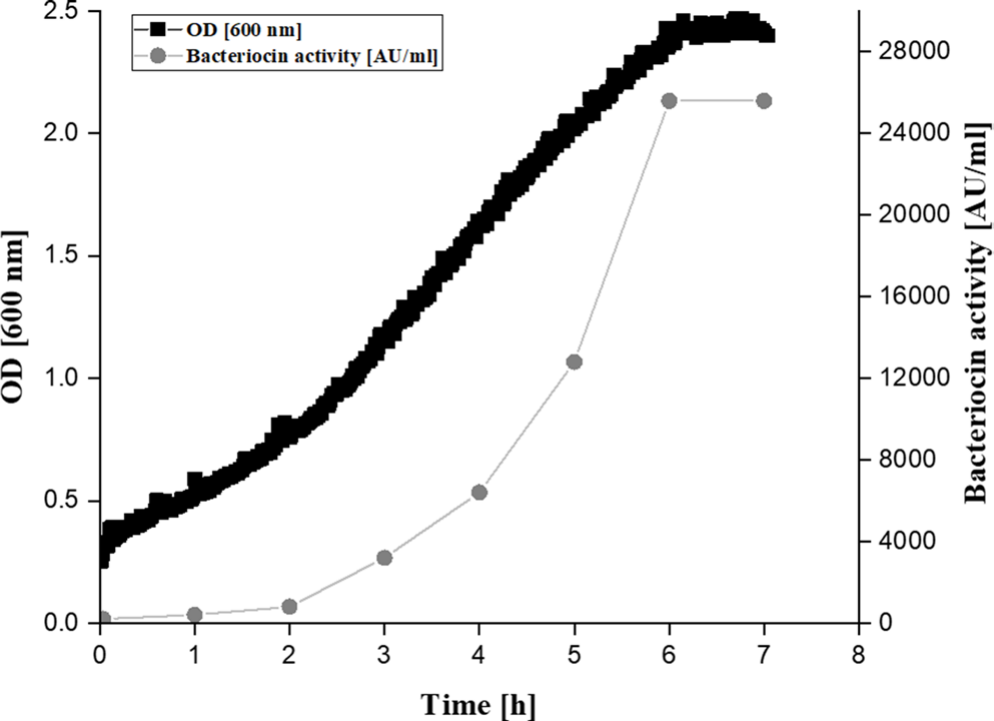
Growth of *L. paragasseri* K7 in 2 L of modified MRS broth in bioreactor (pH 5.75) and production of gassericins K7 (bacteriocin activity, AU/ml)

Fermentation in modified MRS broth (pH 5.75, 37 °C) [27] produced gassericins K7 with typical activity levels of 25,600–51,200 AU/ml in CFS collected after 7 h, with an average of ~ 32,000 AU/ml across all batches. Four production runs were performed, and representative growth and activity profiles are shown in Fig. 1.

After fermentation, the CFS obtained by centrifugation (5 L in total) was used to isolate gassericins. The bacteriocin activity per ml (AU/ml) in the fermentation broth collected after 7 h was typically 25,600 AU/ml or 51,200 AU/ml, and 32,000 AU/ml on average (considering all CFS fractions used for the isolation of bacteriocins, i.e. a total of 5 L).

The purification of gassericins K7 from CFS was based on the protocols of Nilsen et al. [28] and included precipitation with ammonium sulphate, solid phase extraction on the adsorption resin Amberlite XAD-16 and RP-FPLC, which we adapted for the preparative level. Since the type of reversed-phase column (Resource RPC) used in the study by Nilsen et al. [28] showed poor resolution in our system (results not shown), we chose a different column that allowed high flow rates of the highly viscous isopropanol, which is the necessary eluent for Amberlite as it dissolves in acetonitrile. This much longer YMC column showed better resolution and was able to achieve flow rates of up to 7 ml/min during loading.

Starting with 10-times the amount of material (5 L vs. 500 ml CFS) as in the study of Nilsen et al. [28], our aim was to isolate a sufficient amount of active bacteriocins that would allow the study of immunomodulatory activity in vitro on the RAW 264.7 macrophage cell line. We also thoroughly characterised them to investigate whether all four peptides of the gassericin complex of *L. paragasseri* K7 (GasK7A α, GasK7A β, GasK7B α and GasK7B β) were present in the sample pooled from preparative chromatography.

In a typical batch with an initial activity of 51,200 AU/ml, the anti-microbial activity of a fraction obtained after precipitation (800 ml per batch) with ammonium sulphate and resuspension in 20% of the original volume was about 4-times higher, corresponding to 80% recovery of bacteriocin activity. This represents an improvement over the previously reported 42% recovery of gassericins K7 activity [27] and is comparable to the reports on the isolation of GasKT7 [35] and GasGA-3.1 [36] with 92% and 64% recovery, respectively. During the following purification step, solid-phase extraction with Amberlite, we collected four consecutive eluates of 110 ml. In a typical batch, the activity was 102,400 AU/ml in the first two eluates, 51,200 AU/ml in the third eluate and 25,600 in the fourth eluate. The loss of bacteriocin activity during solid phase extraction was 5.5%, and the recovery after ammonium sulphate precipitation and solid phase extraction was 75.6%, which is slightly lower than > 95% after these two steps in the study of Nilsen et al. [28]. After several batches of preparative RP-FPLC, including re-chromatography of the active fractions performed to improve the purity of the samples, we obtained a pooled sample of 241 ml containing 77.11 µg/ml proteins and with an activity of 64,000 AU/mg. The total yield (from 5 L CFS) was therefore 7.43%. The final yields in other studies on gassericins using different isolation approaches were 0.09% for gassericins K7 [27] and 0.7 to 20% for some other gassericins, such as GasKT7, GasGA-3.1 and GasA [37].

**Fig. 2:**
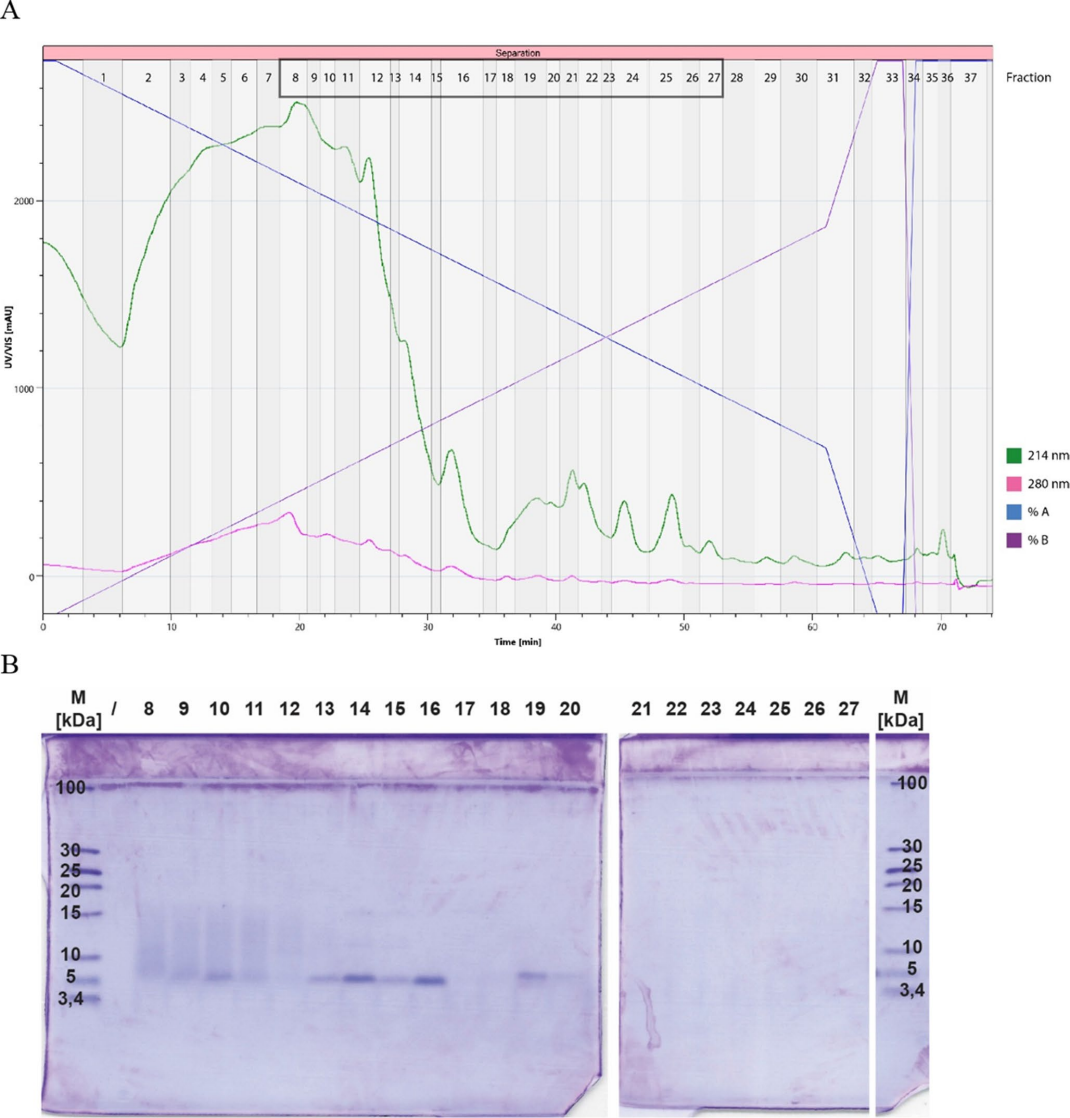
Preparative RP-FPLC chromatogram (214 nm) of a fraction obtained from a cell free supernatant of *L. paragasseri* K7 partially purified by ammonium sulphate precipitation and solid-phase extraction (**A**) and SDS-PAGE of the purified active fractions (**B**). The frame in the chromatogram indicates the fractions with anti-microbial activity

An important problem in the purification was the coloured organic compounds present in the medium. Although most of them were removed by washing the Amberlite with ethanol, these impurities dominated the first 30 min of preparative chromatography and led to a high measured absorption (Fig. 2A). We programmed the preparative RP-FPLC method to remove them as much as possible before eluting the anti-microbial active fractions. These organic compounds, also seen in the chromatogram of Mavrič et al. [27], could probably be at least partially removed from the medium by passing it through Amberlite prior to inoculation with *L. paragasseri*, as suggested by Twomey et al. [38], or by concentrating the CFS by ultrafiltration instead of ammonium sulphate precipitation [27, 38]. However, these options were not investigated in this study, but may be considered in the further optimisation of the isolation of gassericins.

### Characterisation of Gassericins K7

We characterised the active fractions from the final step of RP-FPLC purification by analytical RP-HPLC, SDS-PAGE, antibacterial activity determination and mass spectrometry. Figure 2 shows the typical elution chromatogram of preparative reversed-phase chromatography (A) and SDS-PAGE analysis (B) of the active fractions (8 to 27), showing bands with an apparent molecular mass of about 6 kDa, which are characteristic of gassericins.

We selected several RP-FPLC fractions for mass spectrometry analysis to determine the molecular masses of the isolated peptides. To increase the chances of identifying all four known gassericins of *L. paragasseri* K7, we selected the fractions that fulfilled the following conditions: they eluted at different isopropanol concentrations, showed strong SDS-PAGE bands with the correct molecular weight and had different peaks in the analytical chromatograms. The analyses showed that the molecular mass of two bacteriocins found in samples 3–11 (batch 3, fraction 11) and 5–16 (batch 5, fraction 16) exactly matched to previously characterised gassericins GasK7A α and GasK7B α, respectively (Table 1, Supplementary Fig. 1). GasK7B β was identified in two other fractions in a mixture with GasK7A α and GasK7A β.

**Table 1:** Comparison of molecular masses of peptides determined in purified fractions with mass spectrometry with the theoretical molecular mass of gassericins K7

Sample	Determined molecular mass (Da)	Most likely identity	Theoretical molecular mass (Da)	Discrepancy (Da) and likely reason
3–11	6088 ± 1	GasK7A α	6089	
4–15	4778 ± 1 4776 ± 1 6088 ± 1	GasK7B β (Gly variant) GasK7B β (Ala variant) GasK7A α	4761 4775 6089	+ 16, oxidized Met
5–14	5536 ± 1	GasK7A β (Glu variant)	5521	+ 16, oxidized Met
5–16	5512 ± 1	GasK7B α	5512	
5–19	4778 ± 1 4776 ± 1 6088 ± 1	GasK7B β (Gly variant) GasK7B β (Ala variant) GasK7A α	4761 4775 6089	+ 16, oxidized Met

The determined molecular masses of gassericins are similar to those reported by Mavrič et al. and Nilsen et al. [27, 28]. GasK7A α weighs about 2 Da less than the 6091.5 Da peptide of Nilsen et al. [28], indicating a possible disulfide in our case. The GasK7B α peptides of all three studies are within 1 Da of the theoretical mass (assuming a probable disulfide between Cys26 and Cys30). The molecular mass of GasK7B β determined by Mavrič et al. and Nilsen et al. was within 1 Da (4761.8 Da and 4762.3 Da, respectively) of the theoretical mass (assuming a probable disulfide between Cys21 and Cys32), whereas the molecular mass we determined was 16 Da above the theoretical mass, suggesting oxidation of Met27 [27, 28]. We observed a similar discrepancy of 16 Da between our measured and theoretical mass of GasK7A β, indicating oxidation of Met18. According to analytical RP-HPLC (Supplementary Fig. 2) and SDS-PAGE (Fig. 2B, sample 5–16), GasK7B α (peak eluted at 27.5 min) was the best resolved bacteriocin in this study. This bacteriocin was also previously found to be the most potent among the K7 bacteriocins [28]. The mass spectra of the analysed fractions are shown in Supplementary Fig. 1.

Due to discrepancies between the measured and theoretical molecular mass, the bacteriocins in fractions 4–15 and 5–14 could not be identified with a high degree of certainty. Therefore, a bottom-up MS approach was used in which the proteins were reduced, alkylated and cleaved with trypsin prior to tandem MS analysis. The results are shown in Table 2.

**Table 2:** Identification of gassericins K7 in purified fractions by bottom-up mass spectrometry

Sample	Determined bacteriocin partial sequences	Identity
4–15	*MALKTLEKHE LRNVMGG*NKW GNAVIGAATG ATRGVSWCRG FGPWGMTAC**G** LGGAAIGGYL GYKSN	GasK7B β (Gly variant)*
	*MALKTLEKHE LRNVMGG*NKW GNAVIGAATG ATRGVSWCRG FGPWGMTAC**A** LGGAAIGGYL GYKSN	GasK7B β (Ala variant)
	*MKVLNECQLQ TVVGG*KNWSV AKCGGTIGTN IAIGAWRGAR AGSFFGQPVS VGAGALIGAS AGAIGGSVQC VGWLAGGGR	GasK7A α
	*MIEKVSKNEL SRIYGG*NNVN WGSVAGSCGK GAVM**E**IYFGN PILGCANGAA TSLVLQTASG IYKNYQKKR	GasK7A β (Glu variant)
5–14	*MIEKVSKNEL SRIYGG*NNVN WGSVAGSCGK GAVM**E**IYFGN PILGCANGAA TSLVLQTASG IYKNYQKKR	GasK7A β (Glu variant)*
	*MIEKVSKNEL SRIYGG*NNVN WGSVAGSCGK GAVM**G**IYFGN PILGCANGAA TSLVLQTASG IYKNYQKKR	GasK7A β (Gly variant)
	*MALKTLEKHE LRNVMGG*NKW GNAVIGAATG ATRGVSWCRG FGPWGMTAC**G** LGGAAIGGYL GYKSN	GasK7B β (Gly variant)
	*MKVLNECQLQ TVVGG*KNWSV AKCGGTIGTN IAIGAWRGAR AGSFFGQPVS VGAGALIGAS AGAIGGSVQC VGWLAGGGR	GasK7A α

The signal peptide that is removed after translation to produce the mature bacteriocin is written in italics. The mutation is written in bold. The most abundant peptide detected in each sample is marked with an asterisk (*).

In contrast to Nilsen et al. [28], who failed to isolate GasK7Aβ, we were able to detect all 4 known gassericins of *L. paragasseri* K7 in the purified fractions of CFS: GasK7Aα, GasK7Aβ, GasK7Bα and GasK7Bβ. Furthermore, two genetic variants of each of the β gassericins were identified: GasK7B β had a Gly –› Ala mutation and GasK7A β had a Glu –› Gly mutation. The observed mass discrepancy of about 14 Da between the theoretical and observed mass of the putative GasK7B β in sample 4–15 (Table 2) is presumably due to this mutation from Gly to Ala, which would result in a peptide with a 14 Da higher mass. It is unlikely that this mutation plays a significant role in biological activity as both amino acids are small and hydrophobic. However, the mutation from Glu to Gly in GasK7A β in sample 5–14 replaces an acidic amino acid with a hydrophobic one, which could affect the biological activity of the peptides. The difference in molecular weight due to this mutation would be approximately 71 Da, resulting in a peptide of 5452 Da that was not identified.

Besides the mutations and oxidation of Met, the most common chemical modifications observed in the MS analysis of the tryptic peptides in both samples were the deamidation of Asn and Gln (to Asp and Glu, respectively), both of which caused an increase in molecular weight of about 1 Da. These modifications are often a result of sample processing and MS analysis and are therefore unlikely to be present in the native protein.

The putative class IIc circular gassericin found in the genome of *L. paragasseri* K7 [24] could not be detected in our study or in other studies. We could not detect any activity in a fraction that could not be assigned to one of the four purified gassericins.

### Cell Viability and Anti-inflammatory Activity of Purified Gassericins

To approximate the in vivo situation after digestion of live *L. paragasseri* K7 or a postbiotic preparation containing metabolites and/or inanimate K7 cells, we used a pooled sample containing fractions with different gassericins obtained from different batches for in vitro tests on the cell cultures. We analysed the pooled sample again using RP-HPLC. Supplementary Fig. 3 shows the analytical RP-HPLC chromatogram of the sample with the pooled fractions used for the further experiments.

By analysing a known amount of commercial nisin Z as a standard and comparing the area under the curve of the peaks at 214 nm, we calculated the approximate concentration of gassericins in our bulk sample, which was 77.11 µg/ml, giving a total of 18.58 mg of purified material with a specific activity of 640,000 AU/mg. We used the nisin Z standard as no gassericin standard is available. The pooled fractions were then dried to remove residual organic solvents that could be harmful to the cells.

To test the effect of gassericins K7 in in vitro cultures, a stock solution of 1.51 mg/ml gassericins K7 was prepared from pooled and dried FPLC fractions in 150 mM NaCl buffer and then diluted in DMEM medium to achieve final concentrations of 25, 50, and 100 µg/ml in cell culture. RAW 264.7 macrophages were selected as a representative immune cell type to evaluate the immunomodulatory effects of gassericins K7.

**Fig. 3:**
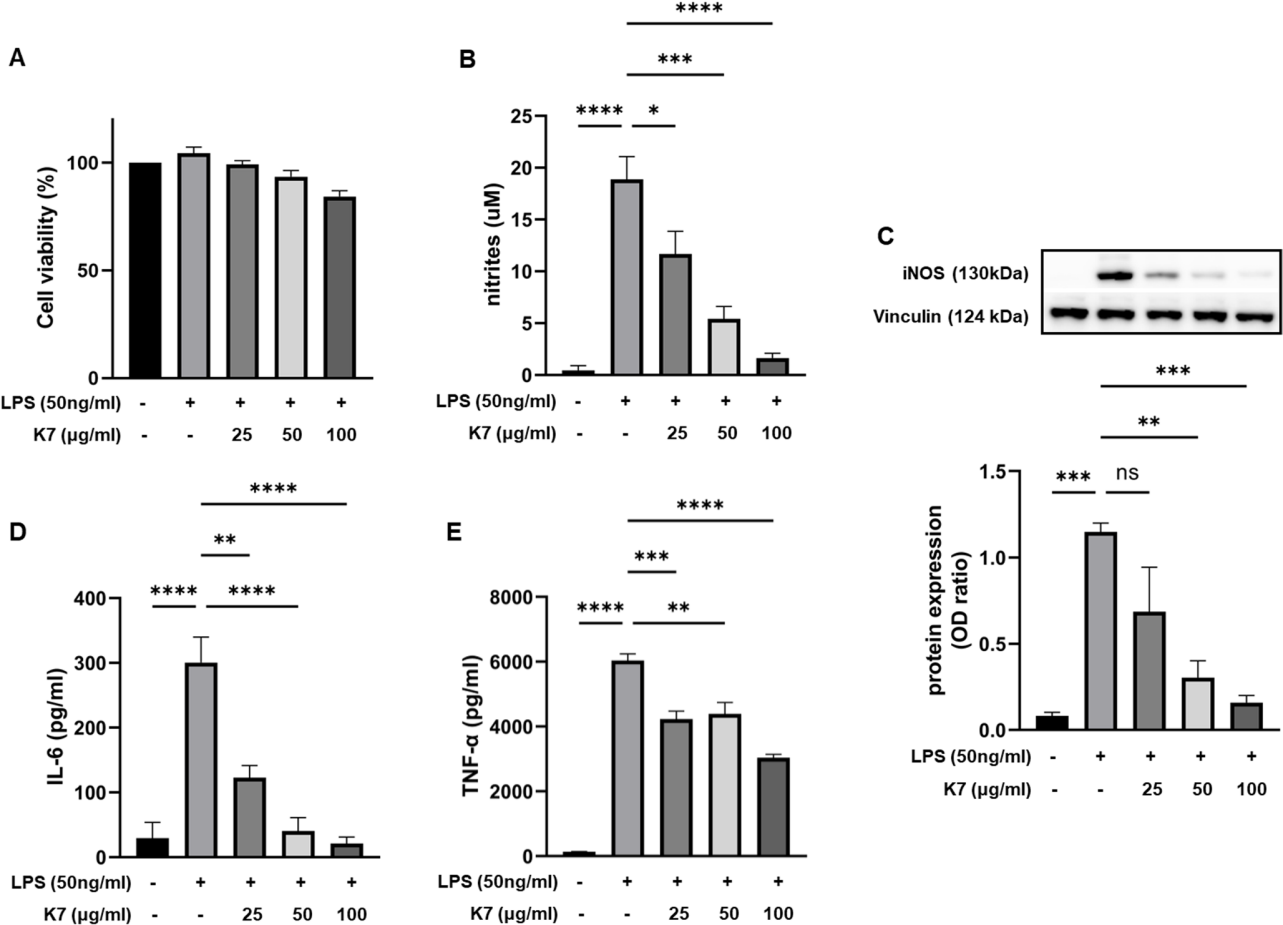
Effect of gassericins K7 on cell viability and immune response. Cell viability (**A**), production of NO (**B**), iNOS expression (**C**), and pro-inflammatory cytokines IL-6 (**D**) and TNF-α (**E**) in RAW 264.7 cells treated with LPS (50 ng/ml) and different concentrations of gassericins K7 were detected after 24 h treatment. Data are expressed as mean ± SD. Statistical analysis was performed using ANOVA followed by Dunnett’s test for multiple comparisons vs. LPS-treated cells (pro-inflammatory markers). Data from the cell viability assay were analysed using a non-parametric Kruskal–Wallis test followed by Dunn’s post hoc test, comparing each treatment group with the control. P value: < 0.1234 (ns), < 0.0332 (*), < 0.0021 (**), < 0.0002 (***), < 0.0001 (****); *n* = 3

The MTT cell proliferation assay revealed that gassericin K7 exerted only minimal, statistically non-significant effects on the viability of RAW 264.7 macrophages across all tested concentrations (Fig. 3A). In general, bacteriocins exhibit low cytotoxicity, but data on their effects on RAW 264.7 cells are limited [16]. The natural isolate of BacSp222 from *Staphylococcus pseudintermedius* strain 222 was not toxic to RAW 264.7 macrophages at a concentration of 1-µM (1 µM BacSp222 ≈ 5.5 µg/ml) [33]. The bacteriocin Ba49 (subtilin) from *Bacillus subtilis* subsp. *spizizenii strain Ba*49, which occurs on the onion *Allium cepa*, showed low toxicity in RAW 264.7 at concentrations up to 6 µM (≈ 35 µg/ml), while at 12.5 µM about 25% of the bacteria lost their viability [39]. High concentrations of synthetic aureocin A53 (*S. aureus* A53) were also found to be toxic to murine macrophage-like cells RAW 264.7. However, similar to our results, the viability of RAW 264.7 was not affected after addition of up to 10 µM aureocin A53 (≈ 58.3 µg/ml). The morphology of RAW 264.7 cells changed significantly after treatment with 20 µM aureocin A53 (20 µM Aureocin A53 ≈ 116.6 µg/ml), and RAW 264.7 cells incubated with a mixture of 10-µM aureocin and IFN-γ exhibited damaged cell membranes, as evidenced by the increased LDH level [40]. To summarise, in line with previous reports showing generally low cytotoxicity of bacteriocins toward RAW 264.7 macrophages, gassericins K7 in our study had minimal effects on cell viability at most concentrations tested. Only the highest dose (100 µg/ml) resulted in a moderate reduction, comparable to the toxicity thresholds observed for BacSp222, Ba49, and aureocin A53 in similar models.

NO production is an important mechanism by which macrophages respond to pro-inflammatory stimuli and is therefore an essential parameter for the assessment of pro- or anti-inflammatory activity. We first examined whether gassericins K7 alone can induce an inflammatory response in RAW 264.7 cells. None of the concentrations tested induced NO production (data not shown), suggesting that gassericins K7 did not elicit a pro-inflammatory effect in these cells. Similar to these results, Smialek et al. reported that BacSp222 (at 1 µM concentration) did not significantly altered NO production in non-stimulated RAW 264.7 cells [33]. We then investigated the ability of gassericins K7 to suppress the pro-inflammatory response induced by LPS from *E. coli* (final concentration: 50 ng/ml). The effect of gassericins K7 on NO production was dose-dependent (Fig. 3B) and was further confirmed by determining the expression of iNOS, the enzyme responsible for NO production in macrophages (Fig. 3C). Since iNOS is a marker (M1) for pro-inflammatory macrophages, we also examined the expression of arginase-I (an M2 marker), but no changes were observed after treatment with gassericins K7 (Supplementary Fig. 4). In contrast, BacSp222 and its variants were shown to increase NO production and strongly induce iNOS expression in RAW 264.7 cells in the presence of IFN-γ, while nisin A had no effect regardless of IFN-γ stimulation [33].

Macrophages are major producers of pro-inflammatory cytokines such as IL-6 and TNF-α. Therefore, we also investigated whether gassericins K7 can reduce their production in LPS-stimulated RAW 264.7 cells. Consistent with its effect on NO production, gassericins K7 showed a dose-dependent attenuation of the LPS-induced inflammatory response (Fig. 3D, E). However, it should be noted that at the highest concentration of GasK7 tested (100 µg/ml), the observed decrease in pro-inflammatory mediator production was at least partially due to non-significanly reduced cell viability. Taken together, our results show that GasK7 alone do not trigger a pro-inflammatory response in RAW 264.7 macrophages, but suppress LPS-induced NO production and pro-inflammatory cytokine secretion in a dose-dependent manner, highlighting their anti-inflammatory potential without shifting macrophage polarization.

There are only a few reports dealing with the effects of other bacteriocins on iNOS-mediated NO production. For example, a similar effect on NO release was observed with the ethyl acetate-soluble supernatant of *Lacticaseibacillus casei* [41], with the cell-free supernatant of lactobacilli cultures [42, 43], or with live or heat-killed bacteriocinogenic lactic acid bacteria, i.e. strains of *Lactiplantibacillus plantarum*, *Pediococcus acidilactici* and *Leuconostoc citreum* [13, 44, 45]. Almost no data has been published on the effect of pure bacteriocins from lactic acid bacteria on RAW 264.7 cells, with the exception of nisin A, a bacteriocin produced by *Lactococcus lactis.* In both quiescent cells and cells stimulated with IFN-γ, nisin A at a concentration of 1 µM did not cause any changes in the production of pro-inflammatory cytokines *(*IFN-γ, IL-12p, IL-17 A, GM-CSF, IL-6, IL-1β, and IL-23) in RAW 264.7 cells [33].

## Conclusions

In this study, we successfully produced, purified, and characterised gassericins K7 from *Lactobacillus paragasseri* K7 using an upscaled purification strategy. The protocol, which involved ammonium sulphate precipitation, Amberlite XAD-16 solid-phase extraction, and preparative RP-FPLC, resulted in improved recovery and yield compared with previous reports. All four known peptides of the gassericin complex (GasK7A α, GasK7A β, GasK7B α, and GasK7B β) were detected, confirming the ability of *L. paragasseri* K7 to synthesise the complete set of gassericins. Mass spectrometry analysis also revealed minor sequence variants and post-translational modifications, suggesting natural diversity within the gassericin K7 complex that may contribute to functional variability.

In functional assays using RAW 264.7 macrophages, the purified gassericins K7 did not show cytotoxicity at any tested concentration. They did not induce nitric oxide (NO) production in non-stimulated cells, indicating an absence of pro-inflammatory activity. In contrast, when macrophages were stimulated with LPS, gassericins K7 significantly suppressed NO production, downregulated iNOS expression, and reduced IL-6 and TNF-α secretion in a dose-dependent manner. These findings highlight the potential anti-inflammatory properties of gassericins K7 and suggest that they may contribute to the immunomodulatory effects of *L. paragasseri* K7 and/or its postbiotic preparations.

Despite these promising results, some limitations should be acknowledged. The study was conducted exclusively in vitro on a single murine macrophage cell line, which limits the extrapolation of results to complex in vivosystems. The precise molecular mechanisms underlying the observed immunomodulatory effects remain to be elucidated, and the biological significance of the identified peptide variants requires further investigation.

Future work should focus on in vivo experiments to confirm the anti-inflammatory and safety profiles of gassericins K7, as well as mechanistic studies on their interaction with immune signalling pathways such as NF-κB, MAPK, or STAT. Structural and functional analyses of individual gassericins, including the identified variants, could further clarify their specific contributions to biological activity. An important consideration for the in vivo application of bacteriocins is their stability during passage through the gastrointestinal tract. Many bacteriocins, especially class II types like pediocin PA-1, are highly sensitive to digestive enzymes (pepsin, trypsin, chymotrypsin) and the acidic environment of the stomach, leading to rapid degradation and loss of antimicrobial activity. Encapsulation in protective matrices (e.g., alginate microcapsules, dual-coated liposomes, pectin-zein beads) can enhance their stability and enable targeted delivery to the colon. Thus, evaluating the stability and bioavailability of gassericins under gastrointestinal conditions, as well as their behaviour in various carrier systems, should be prioritised in future studies.

Overall, this study provides a comprehensive characterisation of the gassericin K7 complex and demonstrates its promising anti-inflammatory potential. These findings support the future application of gassericins K7 as a postbiotic component in functional foods or therapeutics designed to modulate immune responses and promote intestinal health.

## Supplementary Information

Below is the link to the electronic supplementary material.

Supplementary File 1 (DOCX 1.00 MB)

## Data Availability

The datasets used and/or analyzed during the current study are available from the corresponding author on reasonable request.
