# Supplementary material for: Gassericins from Lactobacillus Paragasseri K7: Preparative Isolation and Discovery of Dose-Dependent Anti-Inflammatory Effects
Source: Probiotics Antimicrob Proteins. 2026 Feb 7;18(5):7062–73. doi: 10.1007/s12602-025-10910-x (PMC13369773; doi:10.1007/s12602-025-10910-x)
Supplement: Supplementary file 1 — (DOCX 1.00 MB) [file 12602_2025_10910_MOESM1_ESM.docx]

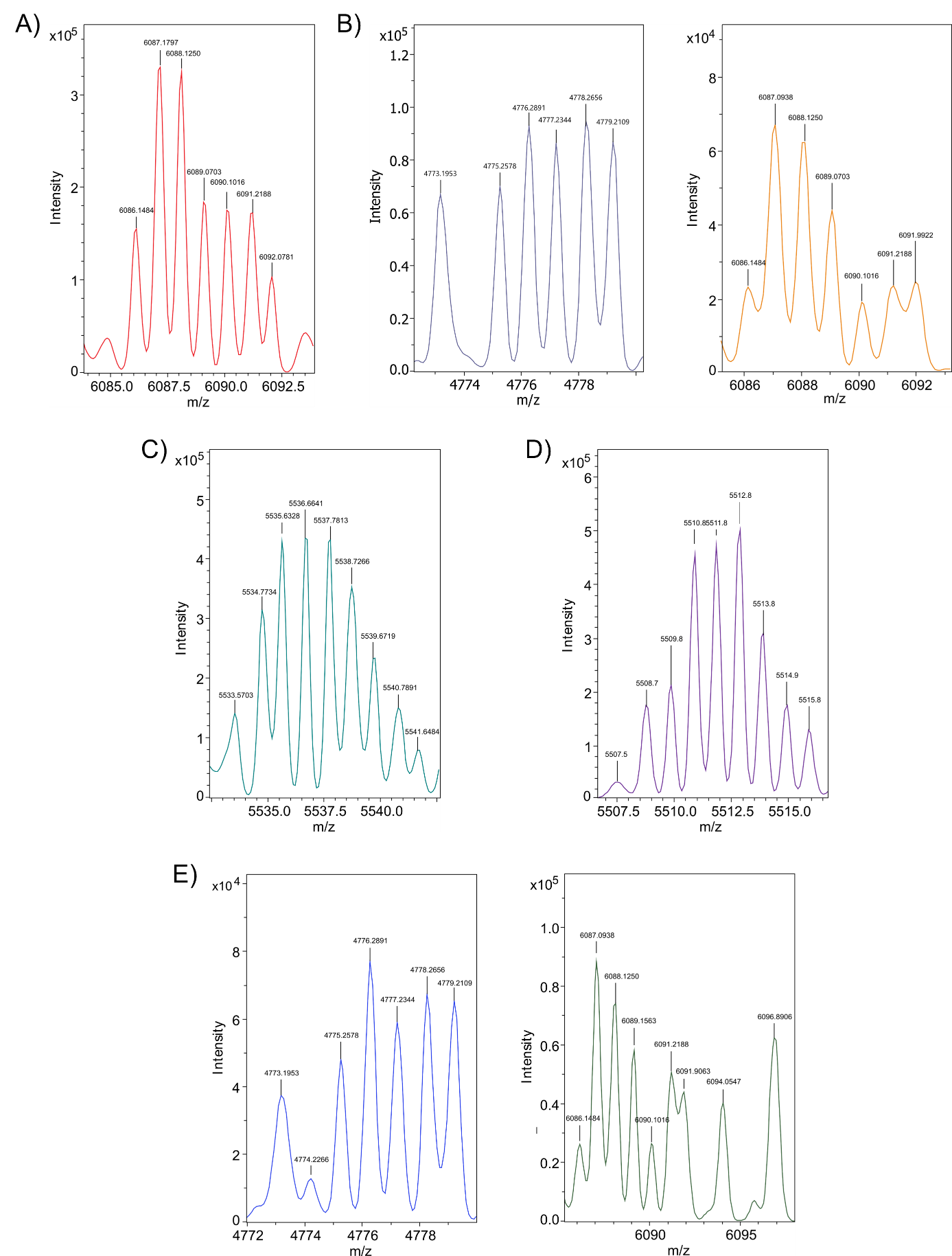


**Suplementary Fig. 1:** Mass spectra of the native gassericins K7 in fractions 3-11 (A), 4-15 (B), 5-14 (C), 5-16 (D) and 5-19 (E). Proteins were in non-reduced state. z = +1

**
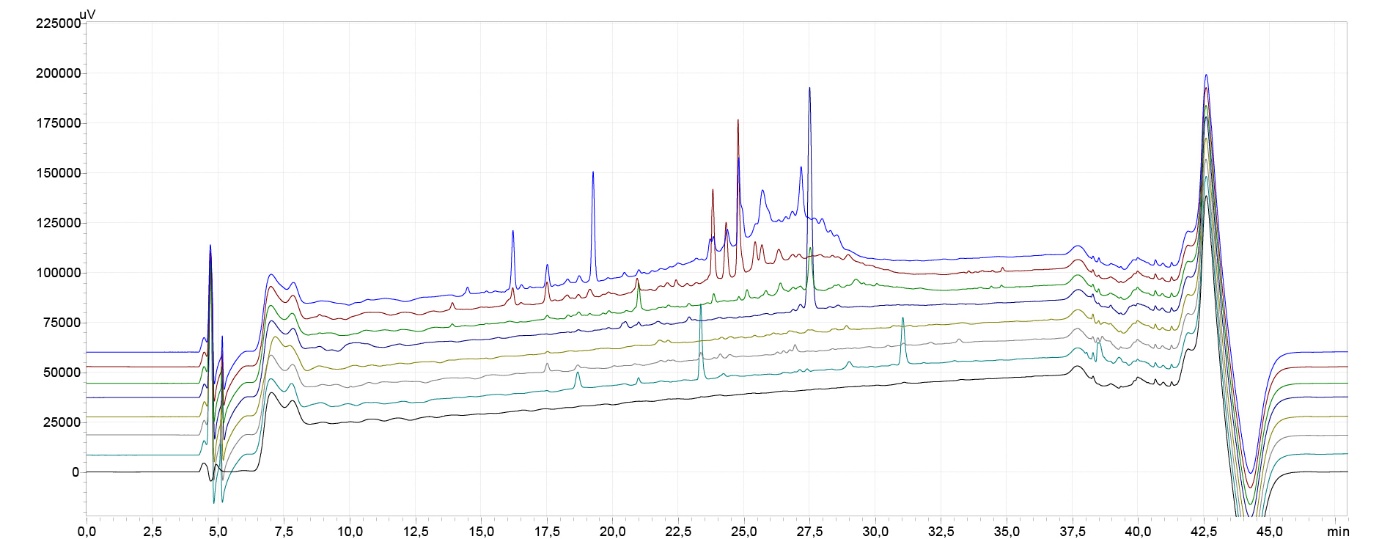
**

**Supplementary Fig. 2.** Analytical RP-HPLC chromatogram (214 nm) of relevant active fractions obtained from a typical RP-FPLC purification of *L. paragasseri* K7. The fractions shown are the same as those in the SDS-PAGE in Fig. 2B. From top to bottom: fractions 13, 14, 15, 16, 17, 18, 19, and the blank run (injection of mobile phase A). GasK7B α in fraction 16 (dark blue) was the best resolved gassericin in this study and the only one for which a peak could be clearly assigned.

**Supplementary Fig. 3.** Analytical RP-HPLC chromatogram of the pooled sample that contains all four bacteriocins of *L. paragasseri* K7.


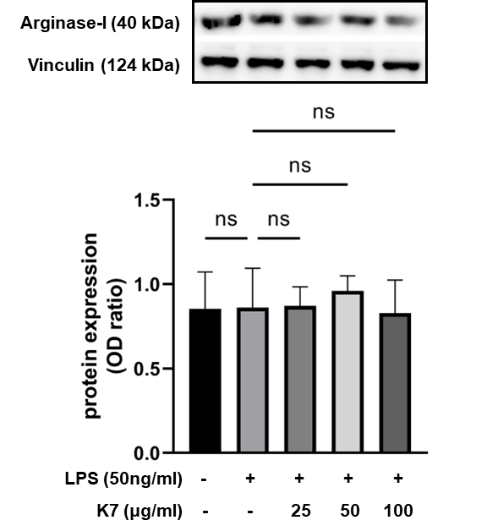


**Supplementary Fig. 4: Effect of gassericins K7 on arginase-I expression.** Arginase-I expression in RAW 264.7 cells treated with LPS (50ng/mL) and different concentrations of gassericins K7 was detected after 24h treatment. Data are expressed as mean ±SD. Statistical analysis was performed using ANOVA was followed by Dunnett’s multiple comparisons test vs. LPS-treated cells; P value: < 0.1234 (ns); n = 3.
